# Regulation of gene expression by miRNA-455-3p, upregulated in the conjunctival epithelium of patients with Stevens–Johnson syndrome in the chronic stage

**DOI:** 10.1038/s41598-020-74211-9

**Published:** 2020-10-14

**Authors:** Mayumi Ueta, Hiromi Nishigaki, Chie Sotozono, Norihiko Yokoi, Katsura Mizushima, Yuji Naito, Shigeru Kinoshita

**Affiliations:** 1 Department of Frontier Medical Science and Technology for Ophthalmology, Kyoto Prefectural University of Medicine, 465 Kajiicho, Hirokoji, Kawaramachi, Kamigyoku, Kyoto, 602-0841 Japan; 2 Department of Ophthalmology, Kyoto Prefectural University of Medicine, Kyoto, Japan; 3 Department of Molecular Gastroenterology and Hepatology, Kyoto Prefectural University of Medicine, Kyoto, Japan

**Keywords:** Biomarkers, Conjunctival diseases, Corneal diseases

## Abstract

To investigate the role of miRNA in the pathogenesis underlying ocular surface complications in patients with Stevens–Johnson syndrome (SJS)/toxic epidermal necrolysis (TEN) in the chronic stage. Using oligonucleotide microarrays, we performed comprehensive miRNA analysis of the conjunctival epithelium of SJS/TEN patients with severe ocular complications (SOC) in the chronic stage (n = 3). Conjunctival epithelium of patients with conjunctival chalasis (n = 3) served as the control. We confirmed the down- and up-regulation of miRNA of interest by quantitative real-time polymerase chain reaction (RT-PCR) assays using the conjunctival epithelium from 6 SJS/TEN with SOC patients and 7 controls. We focused on miRNA-455-3p, which is significantly upregulated in the conjunctival epithelium of the SJS/TEN patients, and investigated its function by inhibiting miR-455-3p in primary human conjunctival epithelial cells (PHCjEs). Comprehensive miRNA expression analysis showed that the expression of 5 kinds of miRNA was up-regulated more than fivefold, and that the expression of another 5 kinds of miRNA was down-regulated by less than one-fifth. There was a significant difference between the SJS/TEN patients and the controls [analysis of variance (ANOVA) p < 0.05]. Quantitative miRNA PCR assay showed that hsa-miR-31* and hsa-miR-455-3p were significantly up-regulated in the conjunctival epithelium of the SJS/TEN patients. Comprehensive gene expression analysis of PHCjEs transfected with the hsa-miR-455-3p inhibitor and quantitative RT PCR assay showed that ANKRD1, CXCL8, CXCL2, GEM, PTGS2, RNASE8, IL6, and CXCL1 were down-regulated by the hsa-miR-455-3p inhibitor. Quantitative RT-PCR, focused on the genes that tended to be up-regulated in SJS/TEN with SOC, revealed that the expression of IL1A, KPRP, IL36G, PPP1R3C, and ADM was significantly down-regulated in PHCjEs transfected with the hsa-miR-455-3p inhibitor. Our results suggest that miRNA-455-3p could regulate many genes including innate immune related genes in human conjunctival epithelium, and that its up-regulation contributes to the pathogenesis on the ocular surface in SJS/TEN patients with the SOC in the chronic stage. Our findings may lead to the development of new treatments using the miRNA-455-3p inhibitor.

## Introduction

Stevens–Johnson syndrome (SJS) is an acute inflammatory vesiculobullous reaction of the skin and mucosa such as the ocular surface, oral cavity, and genitals. Extensive skin detachment and a poor prognosis are called toxic epidermal necrolysis (TEN). Severe ocular complications (SOC) appear in about half of all SJS/TEN patients diagnosed by dermatologists^1^ and cold medicines, including multi-ingredient cold medications and non-steroid anti-inflammatory drugs (NSAIDs) were the main causative drugs of SJS/TEN with SOC^2–6^.

In the acute stage, SJS/TEN with SOC patients present with severe conjunctivitis with corneal and conjunctival erosion, a pseudo-membrane, and skin eruption and erosion. In the chronic stage, ocular surface inflammation persists^7^ and ocular surface complications including dry eye, symblepharon, ankyloblepharon and conjunctival invasion into the cornea may also be present^8,9^ despite healing of the skin lesions.

It is not easy for ophthalmologists to render a differential diagnosis of SJS or TEN when patients present in the chronic stage because the vesiculobullous skin lesions observed in the acute stage have healed. Therefore, the diagnosis of SJS/TEN by ophthalmologists has been based on a confirmed history of acute-onset high fever, serious mucocutaneous illness with skin eruptions, and involvement of at least 2 mucosal sites including the ocular surface^2,4,5,10–12^. Thus, they tend to describe both SJS and TEN with SOC broadly as “SJS”^9^.

MicroRNAs (miRNAs) are small, approximately 22–25 nucleotide-long, endogenous, non-coding RNAs. They regulate gene expression by post-transcriptional interactions with mRNA and are thought to regulate more than one-third of all genes involved in various physiological and pathogenic processes^13^. The role of miRNAs in the development and progression of diseases is becoming recognized^14^.

To investigate the pathogenesis of ocular surface complications in SJS/TEN with SOC in the chronic stage, we performed comprehensive miRNA analysis of the human conjunctival epithelium using GeneChip oligonucleotide microarrays (Affymetrix Inc., Santa Clara, CA). We confirmed the down- and up-regulation of miRNAs of interest by quantitative real-time polymerase chain reaction (RT-qPCR) assays and investigated the function of miRNA-455-3p by transfection with its inhibitor.

## Materials and methods

### Human conjunctival epithelium and primary human conjunctival epithelial cells

This study was approved by the institutional review board of Kyoto Prefectural University of Medicine. All experimental procedures were conducted in accordance with the tenets of the Declaration of Helsinki. Written informed consent was obtained from all patients after they were given a detailed explanation of the purpose of the research and the experimental protocols.

Human conjunctival epithelium was harvested from conjunctival tissue obtained during ocular surface reconstruction for SJS/TEN in the chronic stage or conjunctival chalasis surgery. Samples were immersed overnight at 4 ºC in 1.0 U/ml^−1^ purified dispase (Roche Diagnostic Ltd., Basel, Switzerland)^15^ for comprehensive miRNA analysis, and quantitative miRNA PCR. For transfection with the miRNA inhibitor and RT-qPCR, we cultured primary human conjunctival epithelial cells (PHCjECs) using a modified, previously-described method^16^. Briefly, detached epithelial cells were grown in CnT-prime epithelial culture medium (CELLNTEC, Bern, Switzerland) and 1% antibiotic–antimycotic solution. Cell colonies usually became obvious within 3 to 4 days. After reaching 80% confluence in 7 to 10 days, the cultured PHCjECs were used in the subsequent procedures.

### Comprehensive miRNA analysis

Total RNA was isolated and purified from conjunctival epithelium using the miRNeasy mini kit (Qiagen, Tokyo, Japan) for microarray profiling of miRNAs as recommended by the manufacturer. For all samples the RNA integrity number (RIN) was > 7. Affymetrix GeneChip miRNA 4.0 arrays were used for miRNA profiling. As described by the manufacturer, total RNA was labeled using the FlashTag Biotin HSR RNA labeling kit (Affymetrix Inc., USA). The samples were hybridized on GeneChip miRNA 4.0 arrays (Affymetrix) for 18 h at 48 °C. The arrays were then washed to remove non-specifically bound nucleic acids, stained on a Fluidics Station 450 (Affymetrix), and scanned on a GeneChip Scanner 3000 7G system (Affymetrix).

### Quantitative miRNA PCR

Total RNA was isolated and purified from conjunctival epithelium using the miRNeasy mini kit (Qiagen) as recommended by the manufacturer. For the RT reaction we used ReverTraAce (TOYOBO, Japan). Quantitative miRNA PCR assays were performed on a StepOne plus instrument (Applied Biosystems) according to the manufacturer’s instructions. The primers and probes were purchased from Applied Biosystems.

Universal master mix and the specific primers and probe mixes were included in predesigned Taqman microRNA assays: hsa-miR-455-3p, ID: 002244; hsa-miR-31, ID: 002279; hsa-miR-1285, ID: 002822; hsa-miR-125a, ID: 000448; hsa-mi R-204-3p, ID: 000448; hsa-miR-193b*, ID: 002366 (Applied Biosystems). Normalization was with RNU44 RNA (RNU44, ID: 001094; Applied Biosystems) which served as the internal control.

### Transfection with the miRNA-455-3p inhibitor

The miRNA inhibitor and the control for miRNA-455-3p were purchased from Applied Biosystems. The inhibitor and the negative control were mixed with Lipofectamine RNAiMAX (Invitrogen, Carlsbad, CA) and added for 24 h to PHCjEs at 80% confluence.

### Gene expression analysis of PHCjEs with suppressed miRNA-455-3p

Gene expression profiles were investigated using a high-density oligonucleotide probe array [GeneChip, HumanGene1.0 ST array (Affymetrix)]. Total RNA was extracted with the Qiagen RNeasy kit (Qiagen, Valencia, CA). We used approximately 764,885 probe sets covering more than 28,869 genes. Throughout the process we followed Affymetrix instructions. Scanned microarray images were obtained on a GeneChip Scanner 3000 7G (Affymetrix) using the default settings. Images were visually inspected to detect hybridization artifacts^15^.

### Quantitative RT-PCR

Total RNA was isolated using the RNeasy Mini kit according to the manufacturer’s instructions. For the RT reaction we used ReverTraAce (TOYOBO, Japan). RT-qPCR assays were performed on a StepOne plus instrument (Applied Biosystems) according to the manufacturer’s instructions. The primers and probes were purchased from Applied Biosystems. Quantification data were normalized to the expression of the housekeeping gene GAPDH.

### Data analysis

For microarray analysis we used the ANOVA p-value to record significant differences between SJS patients and the controls. Data from quantitative miRNA PCR assays and RT-qPCR assays were expressed as the mean ± SE and evaluated by the Student’s *t*-test using Microsoft Excel.

## Results

### Comprehensive miRNA analysis

We subjected conjunctival epithelium from 3 SJS/TEN with SOC in the chronic stage- and 3 conjunctival chalasis patients to comprehensive miRNA analysis by microarray.

We found that 52 miRNAs were up-regulated more than fivefold; there was a significant difference between the SJS/TEN patients and the controls [p < 0.05 by analysis of variance (ANOVA)]. As shown in Supplemental Table 1a, of the 52 up-regulated miRNAs, 41 were miR-31 (37 species, 13 of them are 5p and 5 of them are 3p), 8 were miR-72, and one each was identified as miR-7193, miR-455-3p, and miR-1285. We also found that 13 miRNAs were down-regulated by less than one-fifth and that there was a significant difference between the SJS/TEN patients and the controls (p < 0.05, ANOVA) (Supplemental Table 1b). Of the 13 down-regulated miRNAs, 2 were miR-204, 8 were miR-193b, and one each was identified as miR-125a, miR-3535, miR-7847.

### Quantitative miRNA PCR analysis

Based on the result of comprehensive miRNA analysis, we selected human miRNA (hsa-miRNA). Among the 52 up-regulated miRNAs we selected hsa-miR-31*(31-3p), hsa-miR-31-5p, hsa-miR-455-3p, and hsa-miR-1285 (no probe for hsa-miR-72 was available). Among the 13 down-regulated miRNAs we selected hsa-miR-125, hsa-miR-204-3p, and hsa-miR-193b-5p (probes for hsa-miR-3535 and hsa-miR-7847-3p were not available).

**Figure 1:**
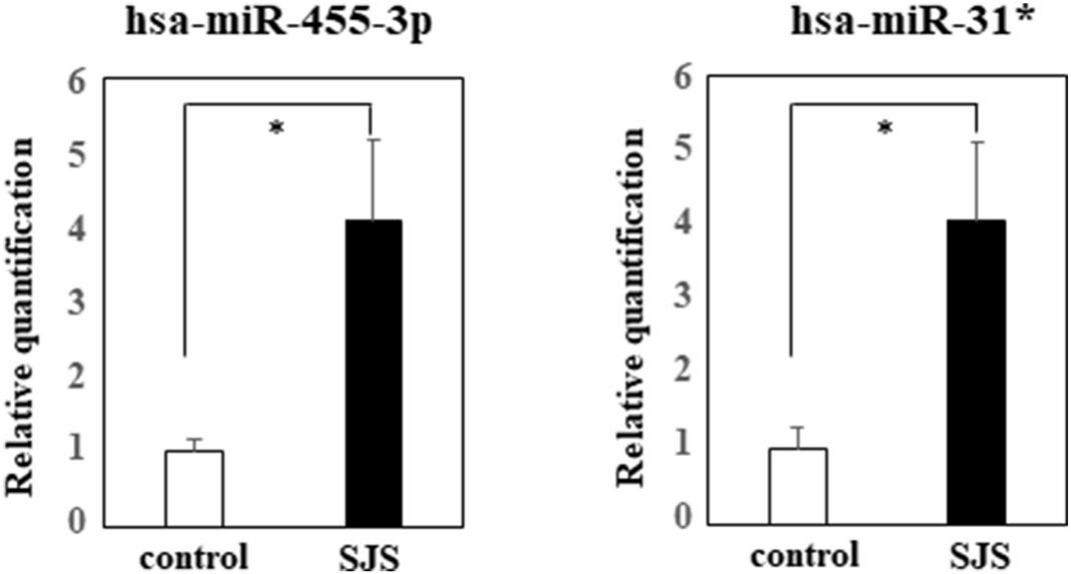
Quantitative miRNA PCR analysis of hsa-miR-455-3p and hsa-miR-31*. Quantification data were normalized to the expression of the internal control, RNU44 RNA. The Y axis shows the increase in specific miRNA over the control samples. Data are the mean ± SEM (controls; n = 7, SJS; n = 6). *p < 0.05.

We subjected conjunctival epithelium from 6 SJS/TEN with SOC in the chronic stage—and 7 conjunctival chalasis patients to quantitative miRNA PCR assay and confirmed that hsa-miR-31* and hsa-miR-455-3p were significantly up-regulated in the conjunctival epithelium of SJS/TEN with SOC (Fig. 1). Between the SJS/TEN patients and the controls we detected no significant difference in hsa-miR-31-5p, hsa-miR-125, hsa-miR-204-3p, hsa-miR-193b-5p, and hsa-miR-1285 (Supplemental Fig. 1).

### Comprehensive gene expression analysis of PHCjEs transfected with the inhibitor of hsa-miR-455-3p miRNA

To examine the function of miRNA we performed comprehensive gene expression analysis of PHCjEs transfected with the hsa-miR-455-3p inhibitor. Quantitative miRNA PCR assay showed that in transfected cells, hsa-miR-455-3p miRNA was down-regulated (Supplemental Fig. 2). Also, the expression of various genes was greatly affected; 49 genes were up-regulated more than threefold and 139 were down-regulated by less than one-third (Supplemental Tables 2a, 2b).

On the other hand, the siRNA of inhibitor of hsa-miR-31* showed less changes of gene expressions; only 2 genes up-regulated more than threefold, and only one gene down-regulated by less than one-third (data not shown). Therefore, we focused on hsa-miR-455-3p, and examined the function for gene expression.

### RT-qPCR analysis of PHCjEs transfected with the hsa-miR-455-3p inhibitor

We selected ANKRD1, CXCL8, CXCL2, GEM, PTGS2, RNASE8, IL6, and CXCL1 that were down-regulated by the hsa-miR-455-3p inhibitor for comprehensive gene expression analysis; ANKRD1, CXCL8, CXCL2, GEM, PTGS2, RNASE8, IL6 were in the top 16 genes and we excluded small nucleolar RNAs, histone clusters, etc.

**Figure 2:**
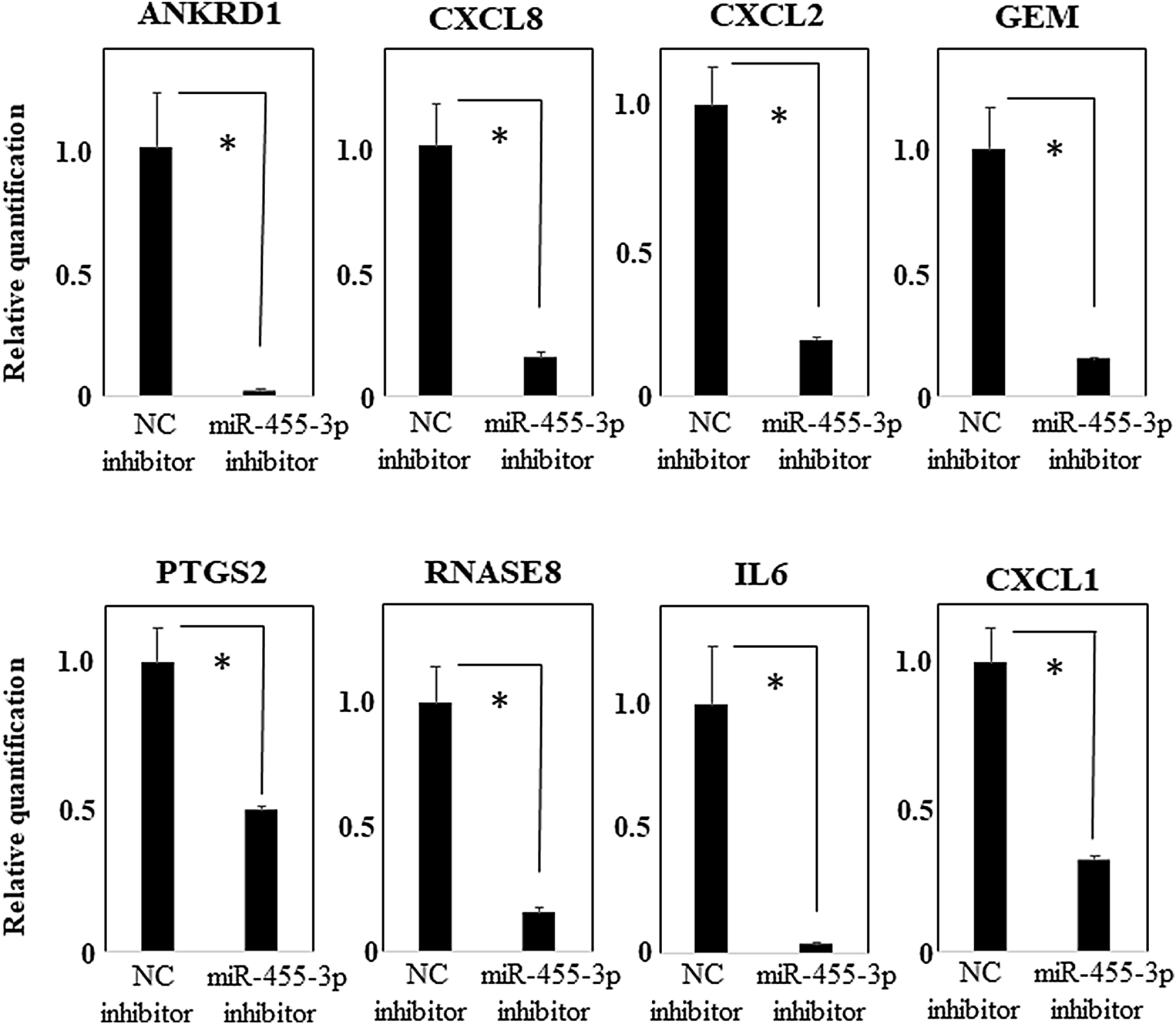
RT-qPCR analysis of PHCjEs transfected with the hsa-miR-455-3p inhibitor. Quantification data were normalized to the expression of the house keeping gene, GAPDH. The Y axis shows the increase in specific mRNA over the control samples. Data are the mean ± SEM (each group n = 4). *p < 0.05.

We investigated their down-regulation by performing RT-qPCR assays. We found that their expression was significantly down-regulated in transfected cells but not in the negative controls (Fig. 2).

**Figure 3:**
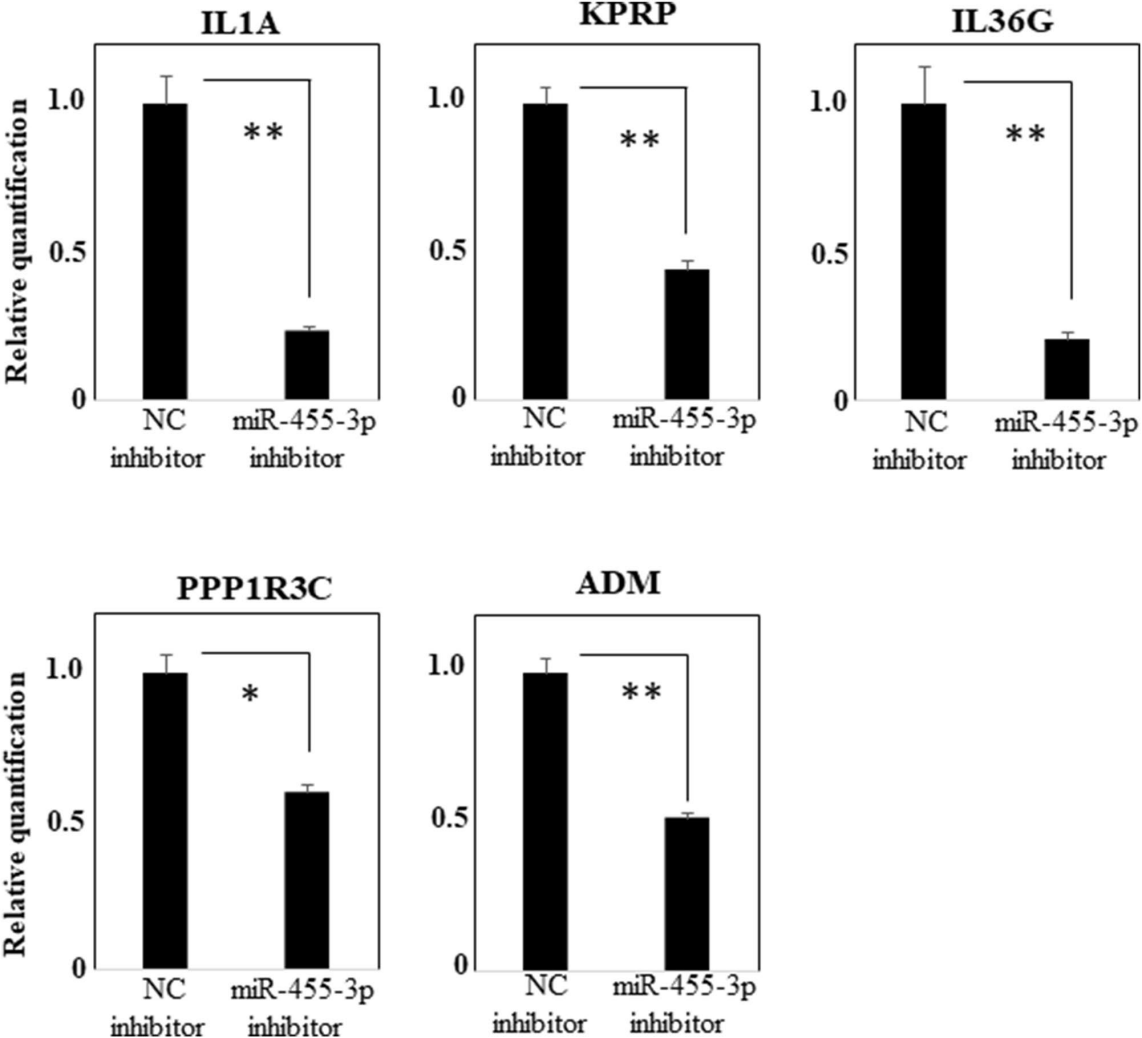
RT-qPCR analysis of the genes which tend to be up-regulated more than threefold in conjunctival epithelium of SJS/TEN with SOC in our previous comprehensive gene expression analysis, and can be down-regulated less than one-half with transfection of hsa-miR-455-3p inhibitor. Quantification data were normalized to the expression of the house keeping gene, GAPDH. The Y axis shows the increase in specific mRNA over the control samples. Data are the mean ± SEM (each group n = 4). *p < 0.05, **p < 0.005.

Elsewhere^15^ we reported that the expression of some genes tended to be up-regulated in the conjunctival epithelium of patients with SJS/TEN with SOC but not in the conjunctival epithelium of the controls. We considered the hypothesis that use of the hsa-miR-455-3p inhibitor may lead to the development of new treatments. Therefore, we selected the genes that in our previously-reported comprehensive gene expression analysis tended to be up-regulated more than threefold in the conjunctival epithelium of SJS/TEN with SOC patients and in our present comprehensive gene expression analysis down-regulated less than one-half after transfection with the hsa-miR-455-3p inhibitor. We found there were 25 such genes (Table 1). RT-qPCR assay confirmed that IL1A, KPRP, IL36G, PPP1R3C, ADM were significantly down-regulated in the presence of the hsa-miR-455-3p inhibitor compared to the negative control in PHCjEs (Fig. 3).

**Table 1:** Results of our previous and present comprehensive gene expression analysis.

Result of our previous comprehensive gene expression analysis of in vivo human conjunctival epithelium							Result of comprehensive gene expression analysis of PHCjEs with has-miR455-3p inhibitor			Gene symbol	Gene description
SJS case1_Signal	SJS case2_Signal	Control 1_Signal	Control 2_Signal	SJS_Avg signal (log2)	Control_Avg signal (log2)	Fold change (linear) (SJS vs. control)	inhNC_Signal	inh455_3p_Signal	inh455_3p/inhNC		
1075.50	111.79	10.62	11.18	8.44	3.45	31.82	1302.82	637.49	0.49	IL1A	Interleukin 1 alpha
2373.56	162.94	32.95	22.89	9.28	4.78	22.64	249.12	68.35	0.27	KPRP	Keratinocyte proline-rich protein
2721.25	57.38	20.30	18.10	8.63	4.26	20.61	80.22	27.45	0.34	IL36G	Interleukin 36, gamma
1255.72	1020.35	107.04	45.95	10.14	6.13	16.14	107.44	41.33	0.38	PPP1R3C	Protein phosphatase 1, regulatory subunit 3C
1403.14	669.83	168.58	83.44	9.92	6.89	8.17	1094.99	391.32	0.36	ADM	Adrenomedullin
489.12	274.64	50.43	40.81	8.52	5.50	8.08	166.21	77.11	0.46	INA	Internexin neuronal intermediate filament protein, alpha
1344.37	211.05	67.88	85.57	9.06	6.25	6.99	858.42	374.40	0.44	RAET1L	Retinoic acid early transcript 1L
225.85	45.91	15.49	14.67	6.67	3.91	6.76	240.41	75.71	0.31	DEFB103A	Defensin, beta 103A
2208.05	237.62	45.91	301.61	9.50	6.88	6.16	1734.57	237.64	0.14	PTGS2	Prostaglandin-endoperoxide synthase 2 (prostaglandin G/H synthase and cyclooxygenase)
229.96	237.24	38.83	45.63	7.87	5.40	5.55	50.67	24.34	0.48	SERPINB9	Serpin peptidase inhibitor, clade B (ovalbumin), member 9
365.23	79.63	34.36	27.58	7.41	4.94	5.54	4912.57	1291.70	0.26	RNASE7	Ribonuclease, RNase A family, 7
842.71	566.70	137.04	134.12	9.43	7.08	5.10	421.65	75.77	0.18	CTGF	Connective tissue growth factor
144.01	60.38	15.35	22.58	6.54	4.22	5.01	745.20	156.61	0.21	DKK1	Dickkopf WNT signaling pathway inhibitor 1
1516.11	196.57	136.99	87.57	9.09	6.78	4.98	186.92	51.03	0.27	HSPA2	Heat shock 70 kDa protein 2
5220.08	3628.07	1250.95	613.56	12.09	9.77	4.97	4999.87	1367.19	0.27	SERPINB2	Serpin peptidase inhibitor, clade B (ovalbumin), member 2
20.02	78.70	8.46	10.11	5.31	3.21	4.29	36.94	9.93	0.27	TNFAIP6	Tumor necrosis factor, alpha-induced protein 6
605.60	337.29	154.66	72.17	8.82	6.72	4.28	201.49	92.51	0.46	UCA1	Urothelial cancer associated 1 (non-protein coding)
1749.92	92.80	165.83	62.12	8.65	6.67	3.97	1757.02	342.16	0.19	NR1D1	Nuclear receptor subfamily 1, group D, member 1
159.87	63.56	20.10	32.57	6.66	4.68	3.94	640.99	135.65	0.21	CCL20	Chemokine (C–C motif) ligand 20
56.56	29.20	11.18	10.38	5.34	3.43	3.77	160.76	67.27	0.42	SNORD114-3	Small nucleolar RNA, C/D box 114-3
1168.76	398.98	186.16	186.53	9.42	7.54	3.66	114.21	46.07	0.40	STX11	Syntaxin 11
153.87	71.00	24.35	36.63	6.71	4.90	3.50	69.37	21.00	0.30	IL20	Interleukin 20
274.93	122.37	35.92	79.02	7.52	5.74	3.44	86.77	37.80	0.44	RND1	Rho family GTPase 1
33.18	22.38	8.45	8.06	4.77	3.04	3.30	25.99	10.65	0.41	THUMPD3-AS1	THUMPD3 antisense RNA 1
65.69	152.47	38.49	27.84	6.64	5.03	3.06	437.17	175.64	0.40	FILIP1L	Filamin A interacting protein 1-like

## Discussion

Our comprehensive miRNA analysis showed that hsa-miR-455-3p and hsa-miR-31* were significantly up-regulated in conjunctival epithelium samples from patients in the chronic stage of SJS/TEN with SOC. Comprehensive gene expression analysis revealed that PHCjEs transfection with the hsa-miR-455-3p inhibitor elicited remarkable changes; 49 genes were up-regulated more than threefold, and 139 were down-regulated by less than one-third.

We focused on hsa-miR-455-3p and examined its function. Using comprehensive gene expression analysis, we found that ANKRD1, CXCL8, CXCL2, GEM, PTGS2, RNASE8, IL6, and CXCL1 were down-regulated in the presence of the hsa-miR-455-3p inhibitor, and we confirmed their significant down-regulation by RT-qPCR assay. Moreover, IL1A, KPRP, IL36G, PPP1R3C, and ADM that tended to be up-regulated in the conjunctival epithelium of SJS/TEN patients, were also significantly down-regulated by the hsa-miR-455-3p inhibitor.

Hsa-miR-455, present on human chromosome 9 at the 9q32 locus, is encoded by the COL27A1 gene (collagen type XXVII alpha-1 chain). The role of miR-455-3p has been implicated in Alzheimer's disease and cancer. Kumar et al.^17^ reported that a high level of miR-455-3p reduced amyloid-β toxicity, suggesting that it may be a biomarker and of therapeutic relevance in Alzheimer's disease. Also, miR-455 has been proposed as a cancer biomarker and it may be involved in the etiology of cancer. Wang et al.^18^ reported that in patients with glioma, miR-455-3p was significantly upregulated and according to Liu et al.^19^, miR-455-3p elicited aberrant up-regulation in various human cancer types and was significantly associated with the lower overall survival of cancer patients. Moreover, Liu et al.^20^ found that miRNA455-3p promotes the invasion and migration of targeting tumor suppressor etoposide-induced 2.4 and suggested that it might be a potential prognostic biomarker and a therapeutic target in patients with triple-negative breast cancer.

Others suggested that miRNA-455-3p functions as an anti-oncogene in human colon cancer by inhibiting the proliferation of cancer cells and by inducing apoptosis^21^. Their subsequent studies revealed that miR-455-3p plays a pivotal role in inhibiting epithelial-mesenchymal transition (EMT) and the TGF-β signaling pathway, and maintains cell differentiation^22^. MiR-455-3p inhibition decreased cell apoptosis and increased the migration, invasion, and EMT of pancreatic cancer cells^23^, and miR-455-3p functions as a tumor suppressor by directly targeting eIF4E in prostate carcinogenesis^24^. According to Hu et al.^25^, miR-455-3p promotes TGF-β/Smad signaling in chondrocytes and inhibits cartilage degeneration by directly suppressing P21-activated kinases and Shao et al.^26^ reported that miR-455-3p-enriched exosomes inhibited the activation of- and cytokine production by lipopolysaccharide-challenged macrophages.

These divergent findings indicate that the function and role of miR-455-3p in these diseases remains to be elucidated. Nevertheless, we show that in the ocular surface, miR-455-3p can regulate the expression of many genes, including genes that are up-regulated on the ocular surface of SJS/TEN with SOC patients. Our findings suggest that miR-455-3p is involved in the pathogenesis of ocular surface lesions in patients with SJS/TEN with SOC. Its inhibition down-regulated ANKRD1, CXCL8 (IL8), CXCL2, GEM, PTGS2, RNASE8, IL6, CXCL1, IL1A, KPRP, IL36G, PPP1R3C, and ADM, suggesting that it plays a role in the regulation of these genes.

Elsewhere^27^ we reported that IL8, IL6, and MCP-1 were remarkably up-regulated in the tears of SJS/TEN with SOC patients in the acute stage, and that even in the chronic stage, IL8 and IL6, but not MCP-1, were significantly up-regulated in their tears^7^. It is possible that miR-455-3p contributes to the up-regulation of IL8 and IL6 in the tears of SJS patients.

Because the tear cytokine profiles are different between in the acute stage^27^ and in the chronic stage^7^, it is possible that there are different in the miRNA profile in the conjunctival epithelium between in the acute stage and in the chronic stage. In this study, we could analyze the miRNA profile in the chronic stage but not in the acute stage, because in the acute stage of SJS/TEN, ocular surface reconstruction surgery might not be performed. The analysis and comparison using acute stage samples might be future subject.

PTGS2 is one of two isozymes of prostaglandin-endoperoxide synthase (PTGS), also known as cyclooxygenase. PTGS is the key enzyme in prostaglandin biosynthesis and it is involved in inflammation^28^. PGE_2_ acts on EP3 and negatively regulates mucocutaneous inflammation of the ocular surface and skin^29,30^. PTGER3, which is gene of EP3, polymorphisms are significantly associated with SJS/TEN with SOC^2^. Interestingly, PTGS2 is necessary for production of PGE_2_. Cold medicines, the principal causative drugs of SJS/TEN with SOC^2–6^ suppress the function of PTGS, suggesting that regulation of PTGS2 by miR-455-3p contributes to the pathogenesis of SJS/TEN with SOC.

CXCL1, an antimicrobial gene, encodes a chemokine of the CXC subfamily and is a ligand for the CXCR3 receptor. CXCL1 binding to CXCR3 elicits pleiotropic effects, including the stimulation of monocytes, the migration of natural killer cells and T-cells, and modulates adhesion-molecule expression^31^. CXCL2 is also an antimicrobial gene; it recruits neutrophils that belong to a chemokine superfamily that encodes secreted proteins involved in immunoregulatory and inflammatory processes^31^.

RNASE8 is a member of the pancreatic ribonuclease family, a subset of the ribonuclease-A superfamily. The gene is expressed prominently in the placenta and exhibits antimicrobial activity against pathogenic bacteria and fungi^32^.

At present, the relationship between CXCL1, CXCL2, RNASE8 and SJS/TEN remains unknown. However, we suggested the possibility of an association between a disordered innate mucosal immune response and SJS/TEN with SOC^9,33,34^. SJS/TEN with SOC patients presented with opportunistic infection of the ocular surface, especially by methicillin-resistant *Staphylococcus aureus* (MRSA) and methicillin-resistant *Staphylococcus epidermidis* (MRSE). Our SJS/TEN with SOC patients suffered persistent inflammation of the ocular surface even in the chronic stage. As their inflammation was associated with colonization by MRSA and MRSE, they developed a hyper-inflammatory reaction against commensal bacteria; the decolonization of MRSA and MRSE may reduce the inflammatory response. On the other hands, it is possible that elevated expression of miR-455-3p in the conjunctival epithelium of SJS/TEN with SOC in the chronic stage is a secondary response to bacterial colonization. Future additional studies are needed to clarify the roles of the miR-455-3p and these antimicrobial genes in the pathogenesis of SJS/TEN with SOC.

ANKRD1 was reported to be localized to the nucleus of endothelial cells. It is induced by IL-1- and TNF-alpha stimulation^35^ and it functions as a transcription factor^36^. GEM belongs to the Rad/Gem/Kir subfamily of Ras-related GTPases whose expression is induced in several cell types upon activation by extracellular stimuli^37^. However, there are no reports on the relationship between ANKRD1 or GEM, and SJS/TEN.

IL1A, KPRP, IL36G, PPP1R3C, and ADM, which tend to be up-regulated in the conjunctival epithelium of SJS/TEN patients, were also regulated by hsa-miR-455-3p. This observation suggests that the regulation of these genes by miR-455-3p contributes to the pathogenesis of SJS/TEN with SOC.

In summary, we found that miRNA-455-3p could regulate many genes and innate immune-related cytokines in the human conjunctival epithelium. The up-regulation of miRNA-455-3p might contribute to the pathogenesis of ocular surface in patients with SJS/TEN with SOC in the chronic stage. Our findings suggest that the miRNA-455-3p inhibitor may lead to the development of new treatments for addressing the ocular complications seen in patients with SJS/TEN with SOC.

## Supplementary information

Supplementary Figure Legends.

Supplementary Figure S1.

Supplementary Figure S2.

Supplementary Table S1.

Supplementary Table S2a.

Supplementary Table S2b.
