# Supplementary material for: Regulation of gene expression by miRNA-455-3p, upregulated in the conjunctival epithelium of patients with Stevens–Johnson syndrome in the chronic stage
Source: Sci Rep. 2020 Oct 14;10:17239. doi: 10.1038/s41598-020-74211-9 (PMC7560850; doi:10.1038/s41598-020-74211-9)
Supplement: Supplementary file 1 — Supplementary Figure Legends. [file 41598_2020_74211_MOESM1_ESM.docx]

**Supplementary Figure Legend**

***Supplementary Figure 1.***

***Quantitative miRNA PCR analysis of other miRNAs.***

Quantification data were normalized to the expression of the internal control, RNU44 RNA. The Y axis shows the increase in specific miRNA over the control samples. Data are the mean ± SEM (controls; n =7, SJS; n =6).

***Supplementary Figure 2.***

***Hsa-miR-455-3p miRNA expression of PHCjEs transfected with the inhibitor***

Quantification data were normalized to the expression of the internal control, RNU44 RNA. The Y axis shows the increase in specific miRNA over the negative control. Data are the mean ± SEM (each group; n =4).
