# Supplementary figures and images for: Regulation of gene expression by miRNA-455-3p, upregulated in the conjunctival epithelium of patients with Stevens–Johnson syndrome in the chronic stage
Source: Sci Rep. 2020 Oct 14;10:17239. doi: 10.1038/s41598-020-74211-9 (PMC7560850; doi:10.1038/s41598-020-74211-9)

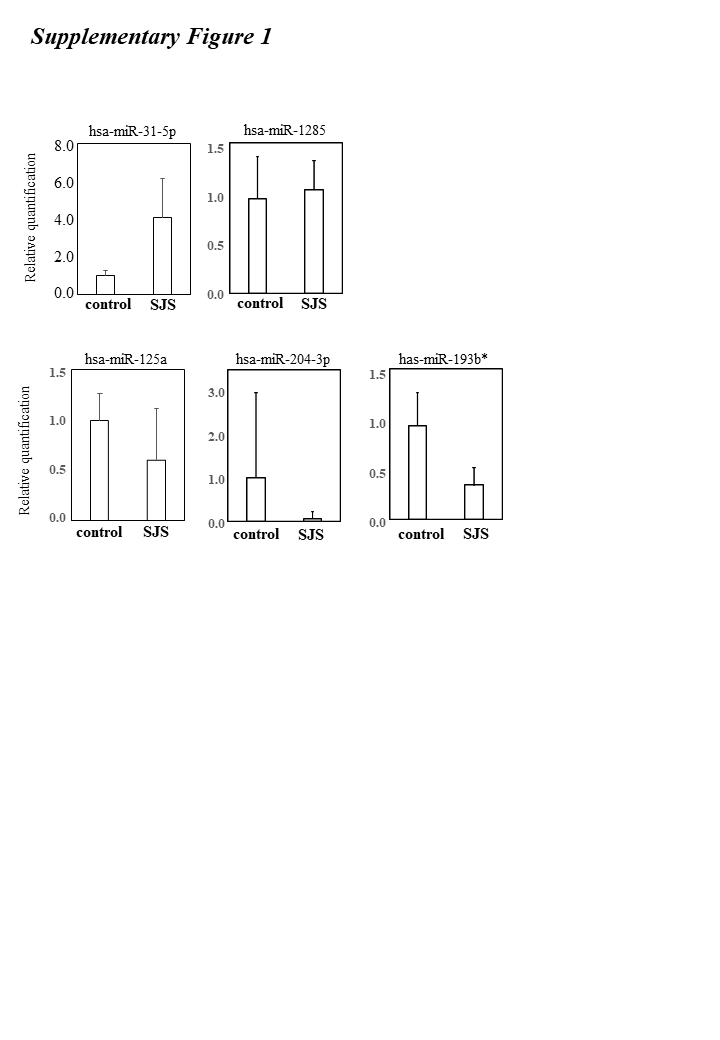

Supplement: Supplementary file 2 — Supplementary Figure S1. [file 41598_2020_74211_MOESM2_ESM.tif]

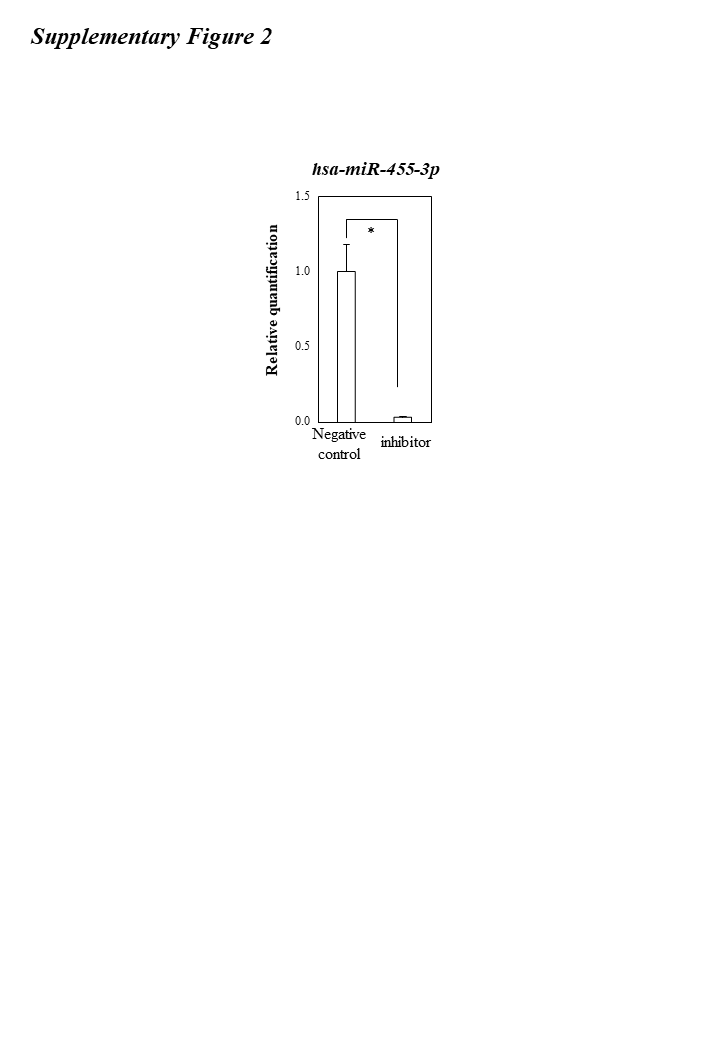

Supplement: Supplementary file 3 — Supplementary Figure S2. [file 41598_2020_74211_MOESM3_ESM.tif]
