# Supplementary material for: Regulation of gene expression by miRNA-455-3p, upregulated in the conjunctival epithelium of patients with Stevens–Johnson syndrome in the chronic stage
Source: Sci Rep. 2020 Oct 14;10:17239. doi: 10.1038/s41598-020-74211-9 (PMC7560850; doi:10.1038/s41598-020-74211-9)
Supplement: Supplementary file 4 — Supplementary Table S1. [file 41598_2020_74211_MOESM4_ESM.docx]

Supplemental Table 1a

The 52 miRNAs that were up-regulated more than 5-fold and were significantly different between the SJS/TEN with SOC patients and the controls [p < 0.05 by analysis of variance (ANOVA)]

| **Probe Set ID** | **Fold Change** | **ANOVA p-value** | **Transcript ID** | **Sequence** |
| --- | --- | --- | --- | --- |
| 20506703 | **42.54** | 0.016 | xtr-miR-31b | GGCAAGAUGCUGGCAAGCU |
| 20507425 | **41.60** | 0.017 | oan-miR-31-5p | AGGCAAGAUGUUGGCAUAGCUGU |
| 20500079 | **32.80** | 0.009 | cel-miR-72-5p | AGGCAAGAUGUUGGCAUAGCUGA |
| 20514579 | **32.80** | 0.009 | bma-miR-72 | AGGCAAGAUGUUGGCAUAGCUGA |
| 20520976 | **32.80** | 0.009 | asu-miR-72-5p | AGGCAAGAUGUUGGCAUAGCUGA |
| 20522783 | **32.80** | 0.009 | hco-miR-72 | AGGCAAGAUGUUGGCAUAGCUGA |
| 20510840 | **31.08** | 0.006 | bfl-miR-31-5p | UGGCAAGAUGUUGGCAUAGCUGU |
| 20530226 | **31.08** | 0.006 | bbe-miR-31-5p | UGGCAAGAUGUUGGCAUAGCUGU |
| 20501968 | **29.05** | 0.011 | gga-miR-31-5p | AGGCAAGAUGUUGGCAUAGCUG |
| 20504976 | **29.05** | 0.011 | xtr-miR-31a | AGGCAAGAUGUUGGCAUAGCUG |
| 20510994 | **29.05** | 0.011 | sko-miR-31 | AGGCAAGAUGUUGGCAUAGCUG |
| 20512053 | **29.05** | 0.011 | crm-miR-72-5p | AGGCAAGAUGUUGGCAUAGCUG |
| 20512147 | **29.05** | 0.011 | ppc-miR-72 | AGGCAAGAUGUUGGCAUAGCUG |
| 20515220 | **29.05** | 0.011 | tgu-miR-31 | AGGCAAGAUGUUGGCAUAGCUG |
| 20529195 | **29.05** | 0.011 | prd-miR-72-5p | AGGCAAGAUGUUGGCAUAGCUG |
| 20522301 | **25.98** | 0.003 | ola-miR-31 | GGGCAAGAUGUUGGCAUAGCUGU |
| 20507808 | **19.35** | 0.017 | odi-miR-31 | AGGCAAGAUGCUGGCAUUGCUG |
| 20500909 | **17.16** | 0.020 | mmu-miR-31-3p | UGCUAUGCCAACAUAUUGCCAUC |
| 20501393 | **17.16** | 0.020 | rno-miR-31a-3p | UGCUAUGCCAACAUAUUGCCAUC |
| 20523210 | **17.16** | 0.020 | cgr-miR-31-3p | UGCUAUGCCAACAUAUUGCCAUC |
| 20528211 | **16.37** | 0.005 | ipu-miR-31 | UGGCAAGAUGUUGGCAUAGCUG |
| 20514749 | **14.84** | 0.003 | aae-miR-31 | UGGCAAGAUGUUGGCAUAGCUGA |
| 20514903 | **14.84** | 0.003 | cqu-miR-31-5p | UGGCAAGAUGUUGGCAUAGCUGA |
| 20528424 | **14.84** | 0.003 | cbn-miR-72a | UGGCAAGAUGUUGGCAUAGCUGA |
| 20513360 | **14.70** | 0.004 | dpu-miR-31 | AGGCAAGAUGUCGGCAUAGCUGA |
| 20510893 | **14.38** | 0.010 | cte-miR-31 | AGGCAAGAUGUUGGCAUAGCU |
| 20510943 | **14.38** | 0.010 | lgi-miR-31 | AGGCAAGAUGUUGGCAUAGCU |
| 20511044 | **14.38** | 0.010 | spu-miR-31 | AGGCAAGAUGUUGGCAUAGCU |
| 20521447 | **14.38** | 0.010 | aca-miR-31-5p | AGGCAAGAUGUUGGCAUAGCU |
| 20519151 | **12.08** | 0.016 | pma-miR-31 | UGGCAAGAUGCUGGCAUAGCC |
| 20505402 | **11.61** | 0.015 | mdo-miR-31-5p | GGAGGCAAGAUGUUGGCAUAGCUG |
| 20505256 | **11.23** | 0.014 | sme-miR-31b-5p | AGGCAAGAUGCUGGCAUAGCUGA |
| 20503335 | **10.04** | 0.015 | mml-miR-31-5p | GGCAAGAUGCUGGCAUAGCUG |
| 20503337 | **10.04** | 0.015 | ptr-miR-31 | GGCAAGAUGCUGGCAUAGCUG |
| 20503338 | **10.04** | 0.015 | ggo-miR-31 | GGCAAGAUGCUGGCAUAGCUG |
| 20503339 | **10.04** | 0.015 | ppy-miR-31 | GGCAAGAUGCUGGCAUAGCUG |
| 20503340 | **10.04** | 0.015 | mne-miR-31 | GGCAAGAUGCUGGCAUAGCUG |
| 20503341 | **10.04** | 0.015 | ppa-miR-31 | GGCAAGAUGCUGGCAUAGCUG |
| 20503336 | **9.98** | 0.006 | mml-miR-31-3p | GCUAUGCCAACAUAUUGCCAUC |
| 20505403 | **9.98** | 0.006 | mdo-miR-31-3p | GCUAUGCCAACAUAUUGCCAUC |
| 20500908 | **9.96** | 0.015 | mmu-miR-31-5p | AGGCAAGAUGCUGGCAUAGCUG |
| 20501392 | **9.96** | 0.015 | rno-miR-31a-5p | AGGCAAGAUGCUGGCAUAGCUG |
| 20523209 | **9.96** | 0.015 | cgr-miR-31-5p | AGGCAAGAUGCUGGCAUAGCUG |
| 20524903 | **9.96** | 0.015 | ssc-miR-31 | AGGCAAGAUGCUGGCAUAGCUG |
| 20508903 | **9.86** | 0.013 | cfa-miR-31 | AGGCAAGAUGCUGGCAUAGCUGU |
| 20500164 | **9.29** | 0.018 | hsa-miR-31-5p | AGGCAAGAUGCUGGCAUAGCU |
| 20504819 | **9.29** | 0.018 | bta-miR-31 | AGGCAAGAUGCUGGCAUAGCU |
| 20514016 | **9.29** | 0.018 | eca-miR-31 | AGGCAAGAUGCUGGCAUAGCU |
| 20504443 | **8.49** | 0.007 | dre-miR-31 | GGCAAGAUGUUGGCAUAGCUG |
| 20526996 | **8.14** | 0.006 | mml-miR-7193-5p | GAGUGCUGGGUUCUGUAGGCCA |
| 20522262 | **7.33** | 0.032 | ola-miR-455-3p | UGCAGUCCAUGGGCAUAUAC |
| 20514333 | **6.02** | 0.000 | ssc-miR-1285 | CUGGGCAACAUAGCGAGACCCCGU |

Supplemental Table 1b

The 13 miRNAs down-regulated less than one-fifth and showed significant differences (ANOVA p-value < 0.05) in the conjunctival epithelium of SJS/TEN with SOC

| **Probe**  **Set ID** | **Fold Change** | **ANOVA p-value** | **Transcript ID** | **Sequence** |
| --- | --- | --- | --- | --- |
| 20523440 | **-17.04** | 0.041 | ggo-miR-125a | UCCCUGAGACCCUUUAACCUG |
| 20529969 | **-13.62** | 0.005 | mmu-miR-3535 | UGGAUAUGAUGACUGAUUACCUGAGA |
| 20529139 | **-9.07** | 0.010 | hsa-miR-7847-3p | CGUGGAGGACGAGGAGGAGGC |
| 20501527 | **-6.39** | 0.001 | rno-miR-204-3p | GCUGGGAAGGCAAAGGGACGUU |
| 20508477 | **-5.19** | 0.001 | mml-miR-204-3p | GGCUGGGAAGGCAAAGGGACGU |
| 20503807 | **-5.09** | 0.010 | hsa-miR-193b-5p | CGGGGUUUUGAGGGCGAGAUGA |
| 20505694 | **-5.09** | 0.010 | mmu-miR-193b-5p | CGGGGUUUUGAGGGCGAGAUGA |
| 20507127 | **-5.09** | 0.010 | oan-miR-193-5p | CGGGGUUUUGAGGGCGAGAUGA |
| 20508461 | **-5.09** | 0.010 | mml-miR-193b-5p | CGGGGUUUUGAGGGCGAGAUGA |
| 20509005 | **-5.09** | 0.010 | cfa-miR-193b | CGGGGUUUUGAGGGCGAGAUGA |
| 20523132 | **-5.09** | 0.010 | cgr-miR-193b-5p | CGGGGUUUUGAGGGCGAGAUGA |
| 20523445 | **-5.09** | 0.010 | ggo-miR-193b | CGGGGUUUUGAGGGCGAGAUGA |
| 20527310 | **-5.09** | 0.010 | mdo-miR-193b-5p | CGGGGUUUUGAGGGCGAGAUGA |
